# Probing Flavonoid-Metal and Membrane Interactions by UV-Vis Spectroscopy: Structural Insights into Bioactivity and Bioavailability

**DOI:** 10.3390/membranes16050179

**Published:** 2026-05-20

**Authors:** Shuangmei Gong, Xiulong Ou

**Affiliations:** Research Center for Preparation and Properties of New Functional Materials, Hanjiang Normal University, Shiyan 442000, China; ouxiulong@hjnu.edu.cn

**Keywords:** flavonoids, UV-Vis absorption spectroscopy, metal ions, membrane–mimetic systems, pKa, antioxidant activity, structure–activity relationship

## Abstract

This study used UV-Vis absorption spectroscopy to investigate the interactions of flavonoids—baicalein (with ortho-dihydroxyl on the A-ring) and apigenin (with 4′-monohydroxyl on the B-ring)—with metal ions (Co^2+^, Ce^4+^) and membrane–mimetic systems (CTAB/SDS micelles, liposomes, vesicles). It revealed how flavonoid spectral properties related to molecular structure and microenvironment. Key findings were as follows: pH affected absorption spectra by altering phenolic hydroxyl protonation. Metal chelation depended on hydroxyl position: baicalein’s A-ring ortho-dihydroxyl formed a stable charge-transfer complex with Cu^2+^. In acidic medium, apigenin reduced Ce(IV) more effectively than baicalein, which contradicted the classic antioxidant role of ortho-dihydroxyl groups. This showed that reaction microenvironments could change hydroxyl reactivity and electron transfer paths. Membrane–mimetic systems (liposomes/vesicles) raised apparent pKa, enhanced solubility and stability. The study first quantified distinct ΔpKa values for different flavonoids (e.g., quercetin vs. baicalein), which were linked to intramolecular H-bonding and membrane preference. Quercetin’s B-ring ortho-dihydroxyl enabled the formation of hydrophobic interfacial anions in nanocarriers under alkaline pH, ensuring high stability. Kaempferol showed sustained leakage. These findings provided a basis for structure-guided flavonoid carrier design, bioavailability, and antioxidant delivery. By integrating reaction microenvironment, membrane interface effects, and carrier stability, this work clarified flavonoid bioactivity mechanisms and supported targeted delivery strategies.

## 1. Introduction

### 1.1. Types, Distribution, and Physiological Activities of Natural Flavonoid Compounds

Natural flavonoid compounds in modern terms broadly refer to a series of compounds constituted by two phenolic benzene rings (the A- and B-rings) connected via a central three-carbon atom. Their basic skeleton in nature is 2-phenyl-chromone, featuring a tricyclic structure formed in a C6–C3–C6 pattern. Common substituents include –OH and –OCH3 [1]. These compounds exist in nature either as free aglycones or as glycosides formed by combination with various sugars. Flavonoid compounds have attracted increasing research interest due to their wide range of pharmacological activities. They are described as beneficial for the treatment of conditions such as diabetes, allergies, cancer, viral infections, and inflammation [2,3]. They can bind to biological macromolecules, enzymes, hormone carriers, and DNA, chelate transition metal ions, catalyze electron transfer, and scavenge free radicals, including superoxide anion radicals.


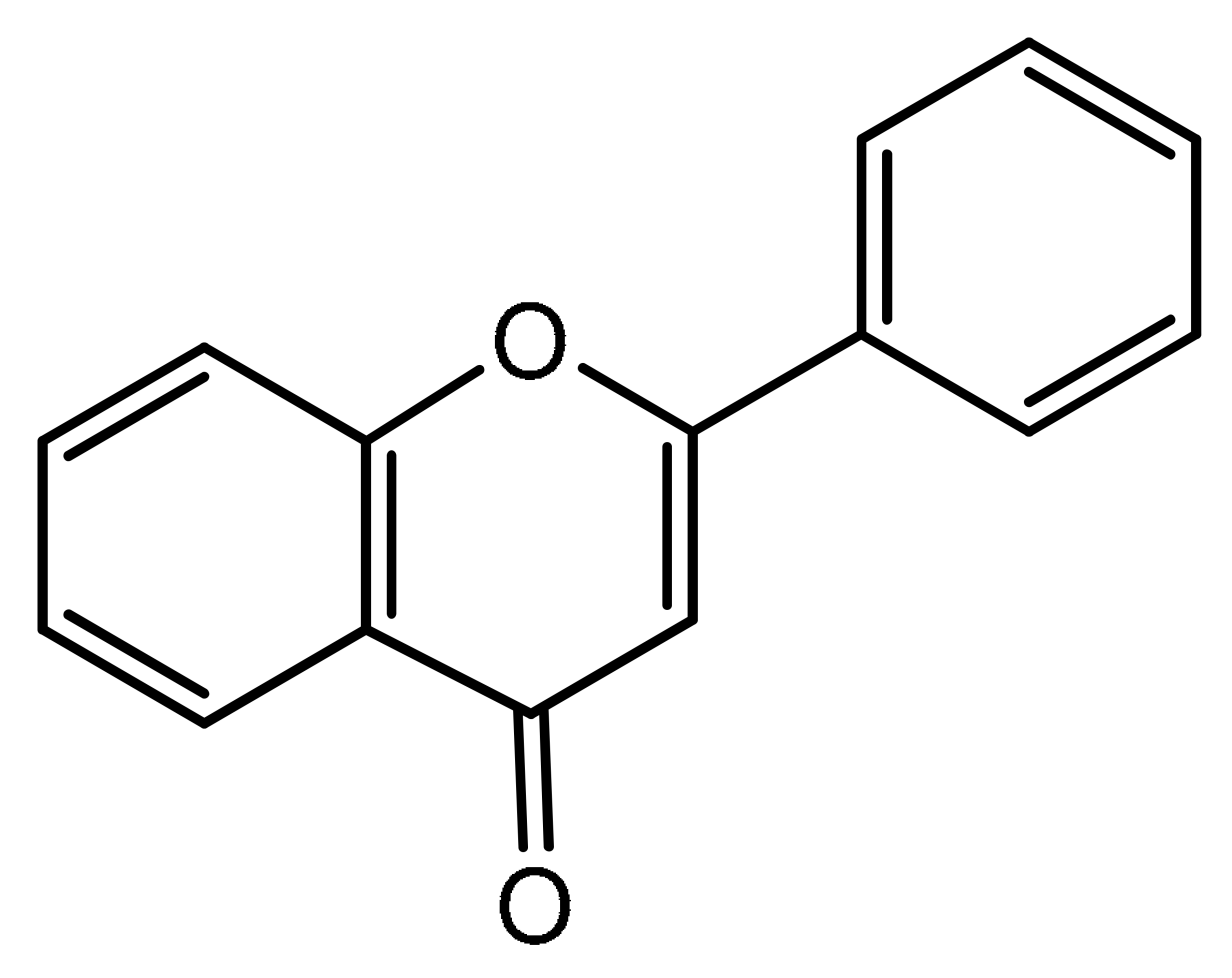

2-phenyl-chromon.

Their excellent antioxidant and free radical scavenging capabilities make these natural polyphenolic substances superior antioxidants. Their antioxidant capacity is closely related to their own structure. Currently, there are numerous reports both domestically and internationally on various pharmacological activities of flavonoids, but comprehensive and reliable summaries of their diverse physiological activities are scarce. In recent years, as functions such as antioxidant activity, inhibition of lipid peroxidation, prevention of cardiovascular diseases, and cancer prevention [4,5,6] have been successively discovered by relevant researchers, activities involving the study, utilization, and development of bioflavonoid substances have been continuously increasing [7].

Flavonoids represent a large group of phenolic plant constituents. To date, over 6000 flavonoid compounds have been identified [8], although a smaller number is more significant from a dietary perspective.

Flavonoids are potent antioxidants in vitro and are thus favorably considered for involvement in protection against cardiovascular diseases. However, the antioxidant effect is one of the mechanisms by which many flavonoids exert their physiological functions [9].

In recent years, many research reports have indicated that flavonoid compounds possess numerous physiological effects, including antioxidant and free radical scavenging activities [10,11], lowering blood lipids and cholesterol [12], protective effects on the cardiovascular system, estrogen-like effects [13], hepatoprotective effects, anti-inflammatory and anti-allergic effects [14], antibacterial and antiviral effects, and antitumor activity [15], among other physiological activities.

### 1.2. Inhibition or Trapping of Free Radicals—Free Radical Traps

Free radicals are active molecules produced by the human body during life activities. Free radicals have certain physiological roles in the human body, but an excess can lead to cellular and tissue organ damage, induce various diseases, and accelerate organismal aging. An excess of free radicals causes oxidative stress within the body, triggering various diseases. If the body’s antioxidants are insufficient at this time and cannot clear the excess free radicals, human health will be compromised [16].

There have also been numerous reports on the antioxidant effects of components containing flavonoids in Chinese herbal medicine. Employing Density Functional Theory (DFT) and Time-Dependent DFT (TD-DFT) calculations, a study systematically investigated the photophysical properties and antiradical activities of six flavonoids, including 5-hydroxyflavone, 3,5-dihydroxyflavone, galangin, kaempferol, quercetin, and myricetin. In assessing antiradical capacity, mechanisms such as Hydrogen Atom Transfer (HAT), Sequential Proton-Loss Electron Transfer (SPLET), and Single Electron Transfer followed by Proton Transfer (SET-PT) were applied. The results revealed that the O4-H4 site of myricetin was the most active site for scavenging free radicals, demonstrating that the formation of intramolecular hydrogen bonds could significantly enhance antioxidant potential [17]. They found that hydroxyl substitutions at the C3′, C4′ positions of the B-ring and the C5, C7 positions of the A-ring were superior to hydroxyl substitution at the C3 position of the C-ring. That is, the difference between flavones and flavonols lies not in whether they are flavones or flavonols, but rather in the hydroxyl substitutions at other positions.

To date, the number and effectiveness of synthetic antioxidants are very limited, and they also possess certain toxicity, teratogenicity, and potential carcinogenicity. Screening for effective and non-toxic free radical scavengers to gradually replace these chemically synthesized antioxidants holds significant practical importance [16]. Many natural products subsequently extracted from plants have been found to possess free radical scavenging capabilities, among which flavonoid compounds are a class with relatively strong activity.

### 1.3. Chelation of Metal Ions

Neurodegenerative diseases are a category of common illnesses that seriously affect human health and frequently occur in elderly patients. Although a very small number of diseases, such as amyotrophic lateral sclerosis (ALS), progress rapidly (within 2–3 years), the onset of the vast majority of neurodegenerative diseases is slow. Conditions like Alzheimer’s disease (AD), Parkinson’s disease (PD), and Huntington’s disease (HD) can have courses lasting 20 years or longer. A shared brain pathology feature of neurodegenerative diseases is the progressive loss of specific cell populations, while different types also exhibit characteristic pathological brain changes. Researchers generally believe that these diseases may share a common mechanism: the abnormal aggregation of specific proteins in central nervous tissue, interactions between redox-active metal ions and these specific proteins, disruption of substances with SOD activity in the brain, increased susceptibility of neurons to oxidation, and ultimately, functional neuronal death. These reactions are caused by abnormal interactions between metals enriched in brain neural tissue and specific vulnerable proteins [18].

It is highly probable that the life-sustaining antioxidant enzyme system is disrupted by abnormal metal ion actions, leading to the occurrence of neurodegeneration. Abnormal interactions between specific tissues and metal ions have become common events in the pathogenesis of neurodegeneration. All these possibilities will be scientifically challenged in the coming years, which will inevitably reposition bio-metals such as zinc, copper, and iron from their current peripheral status in neuroscience to a more important one.

Therefore, metal complexes, especially polynuclear metal complexes, play a crucial role in life processes [19]. The flavonoid structure possesses excellent super-delocalization and a large π-bond conjugated system; the oxygen atoms in flavonoid molecules have strong coordination capabilities; and the spatial structure of flavonoid molecules facilitates the formation of complexes. The involved metal elements are mainly concentrated in copper, zinc, iron, aluminum, and rare earth elements. These metal ions contain empty orbitals that can accept electron pairs provided by ligands, thereby forming complexes [20].

Previous studies [21] have indicated that flavonoid molecules can form analytically valuable complexes with metal ions through the following modes: (1) Chelation involving the hydroxyl group at the 3-position and its adjacent carbonyl group; (2) Chelation involving the hydroxyl group at the 5-position and the carbonyl group at the 4-position; (3) Chelation formation through the combination of two adjacent hydroxyl groups in the B-ring with metal elements. These metal complexes are often colored, making them suitable for spectroscopic detection. Research on these flavonoid metal complexes is primarily motivated by the ability of metal ions to form π-bond conjugated systems with enol-type hydroxyl groups, carbonyl groups, or nitrogen atoms. Some metal complexes also possess their own unique characteristic fluorescence. For example, monohydroxyflavonols exhibit fluorescent characteristics different from other flavonoids. The fluorescence intensity and color of flavonoid metal complex solutions are closely related to the position of hydroxyl groups in the flavonoid molecule, with complexes of flavonoids having hydroxyl groups at the 2, 3, and 5 positions producing the strongest fluorescence.

The connection between transition metal elements and neurodegenerative diseases is so close that the shadow of transition metal elements can be seen behind almost every disease. Therefore, studying the role of transition metal ions in life processes is undoubtedly of great significance for humanity to overcome these diseases. This field also anticipates more in-depth and exciting work.

#### Anti-Lipid Peroxidation

Increasing free radical production and reducing clearance promotes the formation and development of atherosclerosis [22]. Flavonoid compounds, acting as oil antioxidants, function by forming resonance-stabilized radicals with a quinoid structure [23]. In this regard, the stabilizing effect of the C4′-hydroxyl and C4-carbonyl groups is the most important factor for flavones and isoflavones. Additionally, structural factors affecting the antioxidant action of flavonoids include the hydroxyl groups at the C3 and C5 positions [24], which was also found by the author in their study. This is because they can form complexes with metal ions, thereby reducing the initiation role in the autoxidation of oils.

Silymarin is a natural flavonolignan compound, a mixture extracted and refined from the fruits of the Asteraceae plant Silybum marianum, with silybin having the highest content and the strongest activity. Experiments show that this drug can reduce blood cholesterol, clear lipid deposition in liver and kidney tissues, scavenge free radicals, and inhibit lipid peroxidation. It is clinically used to treat hyperlipidemia and fatty liver disease.

### 1.4. Structure and Structure–Activity Relationship of Flavonoid Antioxidant Activity

The remarkable antioxidant and free radical scavenging abilities of flavonoids are determined by their chemical structure. It is widely accepted that the α,β-unsaturated pyranone ring in the flavonoid molecule is key to its diverse biological activities [1].

Generally, a greater number of phenolic hydroxyl groups in a flavonoid molecule provides more hydrogen atoms available for binding with active free radicals. The varying antioxidant activities of flavonoids, specifically their free radical scavenging potency, are primarily related to structural features. These include the substitution pattern and number of phenolic hydroxyls, the presence of double bonds, glycosylation of hydroxyl groups, the 4-carbonyl group, and alkyl/methoxy group substitutions. Furthermore, the p-π conjugation effect of the oxygen atom in the hydroxyl group exerts a strong electron-donating effect, stabilizing the flavonoid radical formed after reaction with an active free radical. The more stable the resulting radical, the stronger the compound’s antioxidant activity.

The presence of ortho-dihydroxyl groups on the B-ring significantly enhances antioxidant activity and is considered the structural basis for highly effective flavonoid antioxidants. Most compounds with strong antioxidant activity in this class possess a 3′,4′-catechol structure, such as quercetin and catechin, with very few exceptions. Ortho-dihydroxy groups readily form more stable ortho-semiquinone anions, which are essential for inhibiting non-enzymatic peroxidation. Among these, the 3′-OH is particularly crucial; the 4′-OH alone contributes little to antioxidant activity [25]. Among all hydroxyl groups, the ortho-dihydroxy groups at the 3′ and 4′ positions on the B-ring are the most important [1]. The diverse physiological activities of flavonoid compounds are largely attributable to the polyphenolic hydroxyl groups present in natural flavonoids. The numerous physiological effects of flavonoids may be related to proton transfer phenomena occurring on their multiple phenolic hydroxyl groups [26,27].

Therefore, analyzing the various physiological and pharmacological actions of flavonoids from the perspective of molecular proton transfer or delocalization is valuable. The antioxidant activity of flavonoids primarily involves the reaction of phenolic hydroxyls with free radicals to form stable semiquinone structures. The strength of this activity is related to the ability of the hydroxyl groups to form stable radical intermediates. The presence of vicinal dihydroxyl groups on the B-ring is a major determinant of antioxidant activity. These adjacent hydroxyls allow one to donate a hydrogen atom, and the resulting structure can form an intramolecular hydrogen bond with the neighboring hydroxyl. This stabilizes the oxidized product, thereby interrupting free radical chain reactions [28,29].

Research on proton transfer in flavonoids is currently very active, attracting increasing numbers of researchers and organizations. Investigative methods extend beyond traditional techniques like synchronous fluorescence spectroscopy and isothermal titration calorimetry (ITC). Researchers are also employing various theoretical analysis tools to examine the impact of the proton transfer process on molecular structure, transition states, electron density, and rotational energy barriers, elucidating the process from multiple angles. These research findings and methodologies will undoubtedly provide essential methodological and theoretical references for subsequent studies, positively contributing to the explanation of flavonoid pharmacological effects. Sengupta and Kasha et al. discovered that 3-hydroxyflavone (3-HF) and fisetin exhibited bright yellow to yellow-green fluorescence in liquid solutions at room temperature, unrelated to the molecules’ UV absorption. They proposed that this yellow fluorescence originated from the excited state of an intramolecular proton transfer tautomer, generated when the parent molecule overcame a double-minimum hydrogen bond potential barrier.

### 1.5. Liposomes, Vesicles and Biomimetic Membrane Systems

Liposomes are spherical vesicles composed of amphiphilic phospholipid bilayers that resemble biological membranes. Each phospholipid consists of a hydrophilic head (formed from phosphate and molecules like choline or serine) and hydrophobic fatty acid tails. In the bilayer, the heads face the aqueous interior and exterior, while the tails align inward, enabling encapsulation of active compounds for targeted delivery. Owing to their biocompatibility and biomimetic structure, liposomes are widely used in research and clinical drug delivery [30,31].

The bilayer exhibits dynamic flexibility, allowing phospholipids to diffuse and release contents in response to environmental changes. This adaptability supports responsive delivery but may compromise stability. Structurally and chemically similar to cell membranes, liposomes can integrate into biological processes, underpinning their pharmaceutical and cosmetic applications.

When a double bilayer (lamellar micelle) curves and seals its edges, it forms a vesicle [32]. Vesicles are enclosed, single- or multi-compartment structures that serve as microreactors, membrane models, and drug carriers [33]. Leveraging the biomimetic and delivery properties of liposomes and vesicles, this study aims to explore the physiological activity and mechanism of flavonoid compounds in a simulated membrane environment.

## 2. Experimental Materials

### 2.1. Reagents

**Figure 1:**
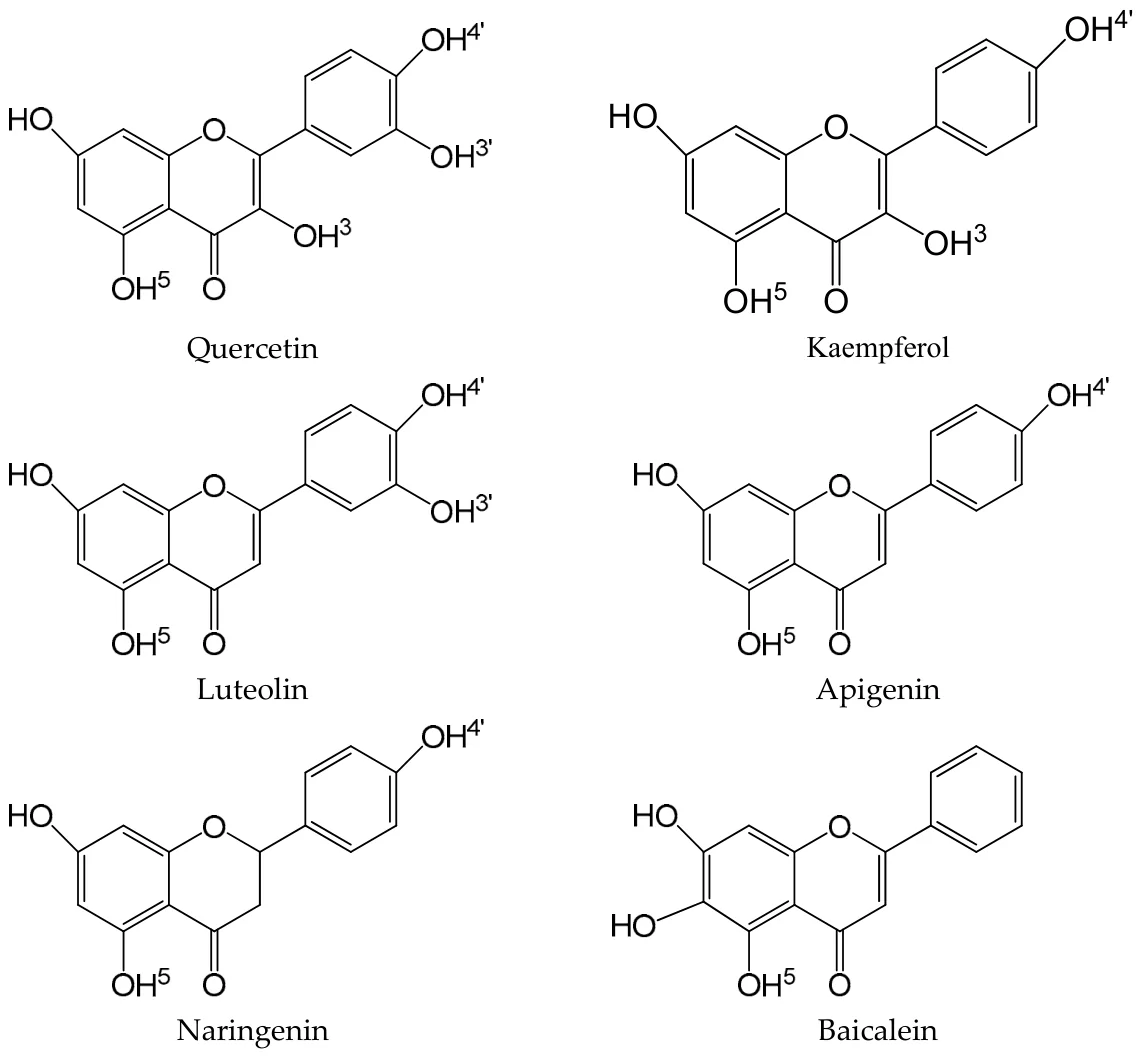
Molecular structures of different flavonoid compounds.

Quercetin, kaempferol, luteolin, naringenin, baicalein, and apigenin (purity 98%, HPLC grade) (Figure 1)were obtained from Nanjing TCM Institute of Chinese Materia Medica. Stock solutions (5 × 10^−3^ mol/L) of each compound were prepared in a mixture of absolute ethanol and water (7:3, *v*/*v*). Ceric(IV) sulfate tetrahydrate (Ce(SO_4_)_2_·4H_2_O) was purchased from Tianjin Fuchen Chemical Reagent Factoy in China. Concentrated sulfuric acid and sodium hydroxide were of analytical reagent grade. 0.04 M Britton-Robinson buffer solution (pH 4.25) was prepared. All other reagents were of analytical grade, and double-distilled water was used throughout.

### 2.2. Instruments and Equipment

Hitachi F-4500 Fluorescence Spectrophotometer (Hitachi Ltd., Japan).

Hitachi UV-3010 UV-Vis Spectrophotometer (Hitachi Ltd., Japan).

XW-80A Vortex Mixer (Shanghai Jia Peng Technology Co., Ltd., China).

KQ-100 Ultrasonic Cleaner (Kunshan Ultrasonic Instrument Factory, China).

DZF-6020 Vacuum Drying Oven (Shanghai Boxun Industrial Co., Ltd., China).

Electronic Balance (d = 0.0001 g) (Sartorius Instrument Systems Co., Ltd., Beijing, China).

DHG-9140A Electric Thermostatic Blast Drying Oven.

Refrigerator (Haier Group, China).

### 2.3. Experimental Methods

#### 2.3.1. Preparation of Stock Solutions

A solvent mixture of absolute ethanol and double-distilled water (7:3, *v*/*v*) was used throughout. Stock solutions (5 × 10^−3^ mol/L) of quercetin (0.0169 g), kaempferol (0.0143 g), luteolin (0.0143 g), and naringenin (0.0136 g) were prepared by dissolving each accurately weighed compound in the ethanol/water mixture, vortexing, and diluting to 10 mL in volumetric flasks. The solutions were stored refrigerated.

Due to the hydrolytic tendency of Ce^4+^ in aqueous media, concentrated sulfuric acid was added during preparation to suppress hydrolysis and ensure complete dissolution [34]. The hydrolysis equilibria are as follows:

Ce^4+^ + H_2_O ⇌ Ce(OH)^3+^ + H^+^, K_1_ = 0.2

Ce(OH)^3+^ + H_2_O ⇌ Ce(OH)_2_^2+^ + H^+^, K_1_ = 0.16

Ce^4+^ solution (2.0 × 10^−3^ mol/L) was prepared by dissolving 0.0809 g of Ce(SO_4_)_2_·4H_2_O in 25 mL of distilled water, followed by the slow addition of 17 mL of concentrated sulfuric acid under stirring. After thorough mixing and cooling to room temperature, the solution was transferred to a 100 mL volumetric flask and diluted to volume with distilled water.

Britton-Robinson (B-R) buffer stock solution (1/25 mol/L mixed acid) was obtained by combining 2.71 mL of 85% orthophosphoric acid, 2.36 mL of glacial acetic acid, and 2.47 g of boric acid, then diluting to 1 L with distilled water. A 0.2 mol/L NaOH solution was prepared by dissolving 8.0 g of NaOH in distilled water to a final volume of 1 L. The working buffer (pH 4.25) was prepared by mixing appropriate volumes of the B-R stock solution and the 0.2 mol/L NaOH solution.

Na_2_SO_4_ solution was prepared by dissolving 35.50 g of Na_2_SO_4_ in water, quantitatively transferring to a 250 mL volumetric flask, slowly adding 54.64 mL of concentrated sulfuric acid, mixing, cooling, and diluting to volume.

0.01 mol/L Co^2+^ stock solution was prepared by dissolving 0.2379 g of CoCl_2_·6H_2_O in distilled water and diluting to 100 mL.

Vesicles were prepared by mixing Tween 80, PEG 6000, Span 80, and H_2_O in a mass ratio of 1.000:0.025:0.30:0.500, followed by vortexing. The blend was then mixed with an equal volume of water and sonicated for 30–85 min.

Liposomes were prepared by dissolving 500 mg of egg phosphatidylcholine (EPC) in 5 mL of an organic solvent mixture (chloroform/methanol, 9:1 *v*/*v*). The organic solvent was removed by rotary evaporation at 30 °C, yielding a thin lipid film on the flask wall. Residual solvent was eliminated by purging with N_2_ for 10 min. Subsequently, 50 mL of pH 7.4 buffer was added under N_2_ protection, and the mixture was incubated at 40 °C for 10 min, vortexed for 1 min, and then subjected to intermittent ultrasonication in an ice bath under continuous N_2_ for 15 min. The resulting suspension was centrifuged at 3000 rpm for 20 min at 2–4 °C. The supernatant, a translucent 10 mg/mL EPC liposome suspension, was collected and stored under N_2_ at 4 °C for use within three days [35].

#### 2.3.2. UV Absorption Spectrometry Signal Detection

UV absorption spectra were recorded over the wavelength range of 200–500 nm using the corresponding buffer as reference. Two experimental setups were employed.

In the first setup, the Ce^4+^ concentration was held constant at 2.0 × 10^−4^ mol/L, while the flavonoid concentration varied from 0.5 × 10^−6^ to 7.5 × 10^−6^ mol/L. This was achieved by sequentially adding 2 µL aliquots of 5.0 × 10^−4^ mol/L flavonoid stock solutions to a quartz cuvette containing 1.8 mL of 0.04 M Britton-Robinson buffer (pH 4.25) and 200 µL of 0.002 mol/L Ce^4+^ solution. After each addition, the mixture was allowed to stand for 5 min before scanning.

In the second setup, each of the six flavonoid compounds was maintained at a fixed concentration of 2.0 × 10^−5^ mol/L, while the Ce^4+^ concentration ranged from 4.0 × 10^−6^ to 4.8 × 10^−5^ mol/L. To a quartz cuvette containing 2 mL of pH 4.25 B-R buffer and 8 µL of 5 × 10^−3^ mol/L flavonoid stock solution, 4 µL aliquots of 0.002 mol/L ceric sulfate solution were sequentially added, with a 5 min equilibration period after each addition. Spectra were recorded over 220–500 nm.

Stock solutions of natural flavonoids (e.g., quercetin) were prepared at 5.0 × 10^−4^ mol/L in absolute ethanol/water (7:3, *v*/*v*). Extracted samples were similarly prepared as 2 mg/mL solutions in the same solvent mixture.

The detection reagent was formulated to contain 0.3 mol/L H_2_SO_4_, 1.0 mol/L Na_2_SO_4_, and 2.0 × 10^−4^ mol/L Ce(IV). It was prepared by combining 1706 µL of Na_2_SO_4_ solution (obtained by dissolving 35.50 g of Na_2_SO_4_ in water, adding 54.64 mL of concentrated H_2_SO_4_, cooling, and diluting to 250 mL), 94 µL of H_2_O, and 0.2 mL of Ce(IV) solution (2.0 × 10^−3^ mol/L Ce(SO_4_)_2_ prepared by dissolving 0.0809 g of Ce(SO_4_)_2_·4H_2_O in 25 mL distilled water and 17 mL concentrated H_2_SO_4_, then diluting to 100 mL).

For detection, the absorption spectrum of 2 mL of the detection reagent was recorded over 220–500 nm using distilled water as reference. Subsequently, test compound or sample solutions were added in 2 µL aliquots (total added volume <1/100 of the reagent volume). After each addition and a 10 min equilibration period, the spectrum was recorded. All measurements were performed in triplicate at room temperature.

#### 2.3.3. UV Spectroscopic Detection of the Interaction Between Flavonoid Compounds and Metal Ions

To a cuvette containing 2 mL of double-distilled water, 8 μL of flavonoid stock solution was added, yielding a final flavonoid concentration of 2.0 × 10^−5^ mol/L. The UV absorption spectrum was recorded over 200–500 nm. Then, 2 μL aliquots of 0.01 mol/L metal ion solutions (Co^2+^ or Ce^4+^) were sequentially added, and after each addition, the spectrum was recorded.

## 3. Results and Discussion

This study systematically investigated the spectral behaviors of two structurally related flavonoid compounds—baicalein (Bai) and apigenin (Api)—in different chemical environments and their interactions with biologically relevant metal ions, oxidants, and membrane–mimetic systems using UV-Vis absorption spectroscopy. The aim was to elucidate their structure–activity relationships at the molecular level.

### 3.1. pH-Dependent Spectral Behavior: Ionization States and Conjugation Effects of Baicalein and Apigenin

**Figure 2:**
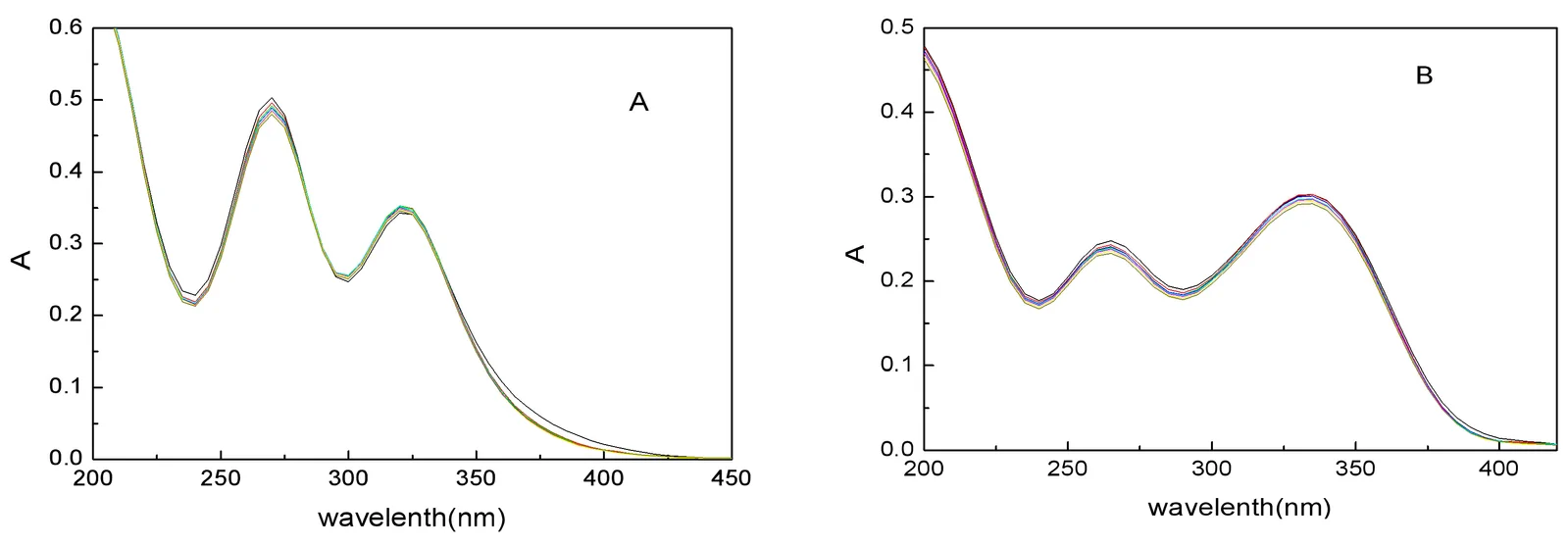
UV-vis spectra of (**A**) baicalein, (**B**) apigenin and H_2_SO_4_ with different molar ratio. C_(flavonoids)_ = 4.0 × 10^−5^ mol/L, 0–9: C(H_2_SO_4_) = 0, 1.0 × 10^−5^, 1.5 × 10^−5^, 2.0 × 10^−5^, 2.5 × 10^−5^, 3 × 10^−5^, 3.5 × 10^−5^, 4.0 × 10^−5^, 4.5 × 10^−5^, 5.0 × 10^−5^ mol/L.

**Figure 3:**
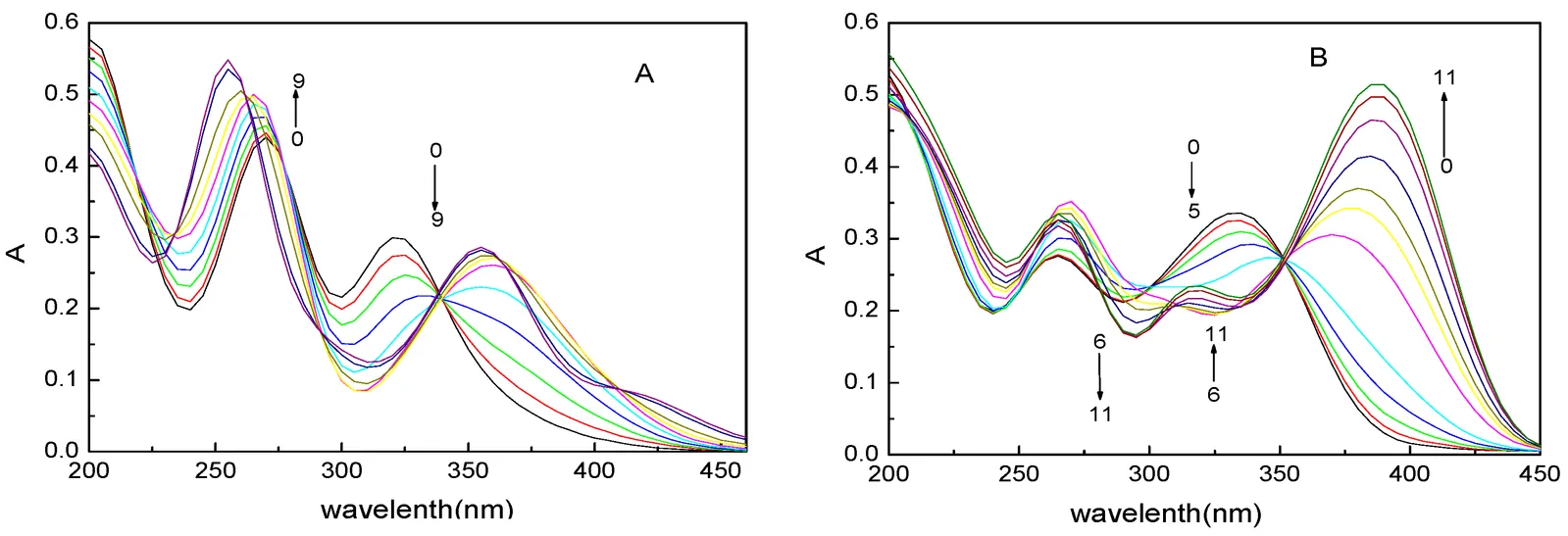
UV-Vis spectra of fixed concentration of flavonoids (2.0 × 10^−5^ M) in the presence of NaOH (0.01 mol/L) of varying concentration (0 M to 9 × 10^−5^ M). (**A**) Baicalein, (**B**) Apigenin.

Exploration of the acid-base characteristics of Bai and Api began with monitoring of their UV absorption spectra under conditions of gradual acidification (Figure 2) and alkalinization (Figure 3). Both compounds exhibited significant and reversible pH-dependent spectral changes, closely related to the protonation/deprotonation processes of their phenolic hydroxyl groups.

Under acidic conditions (with the addition of H_2_SO_4_), as shown in Figure 2, at low concentrations of H_2_SO_4_ (≤5.0 × 10^−5^ mol/L), the UV-Vis absorption spectra of baicalein and apigenin showed no significant change compared to those in the absence of acid, indicating that the conjugated structure and electronic transitions of the flavonoid molecules were not significantly affected within this acidity range.

In contrast, under alkaline conditions (with the addition of NaOH), the spectra showed a significant red shift along with a hyperchromic effect. The long-wavelength absorption band of Api could red-shift from 336 nm to above 380 nm, a greater change than that of Bai (which shifted from 320 nm to above 350 nm). This indicated the gradual deprotonation of phenolic hydroxyl groups, forming phenolate anions. The phenolate anions formed by deprotonation had strong electron-donating capabilities. They greatly enhanced the electron delocalization of the molecular π system through p-π conjugation, effectively narrowing the HOMO-LUMO energy gap [36].

Notably, under alkaline conditions, Api (with a 4′-OH on the B ring) exhibited a larger red shift magnitude and a higher final absorption wavelength than Bai (which has no hydroxyl group on the B ring). This sensitive pH-responsive property suggested that flavonoids might exist and function in different ionization states within physiological compartments of varying pH (such as gastric fluid and cytosol).

### 3.2. Metal Chelation: Spectral Evidence and Elucidation of Coordination Modes

An important biological function of flavonoid compounds is their metal chelation capability. The present study investigated the interactions of Bai and Api with the metal ions Co^2+^ and Ce^4+^.

**Figure 4:**
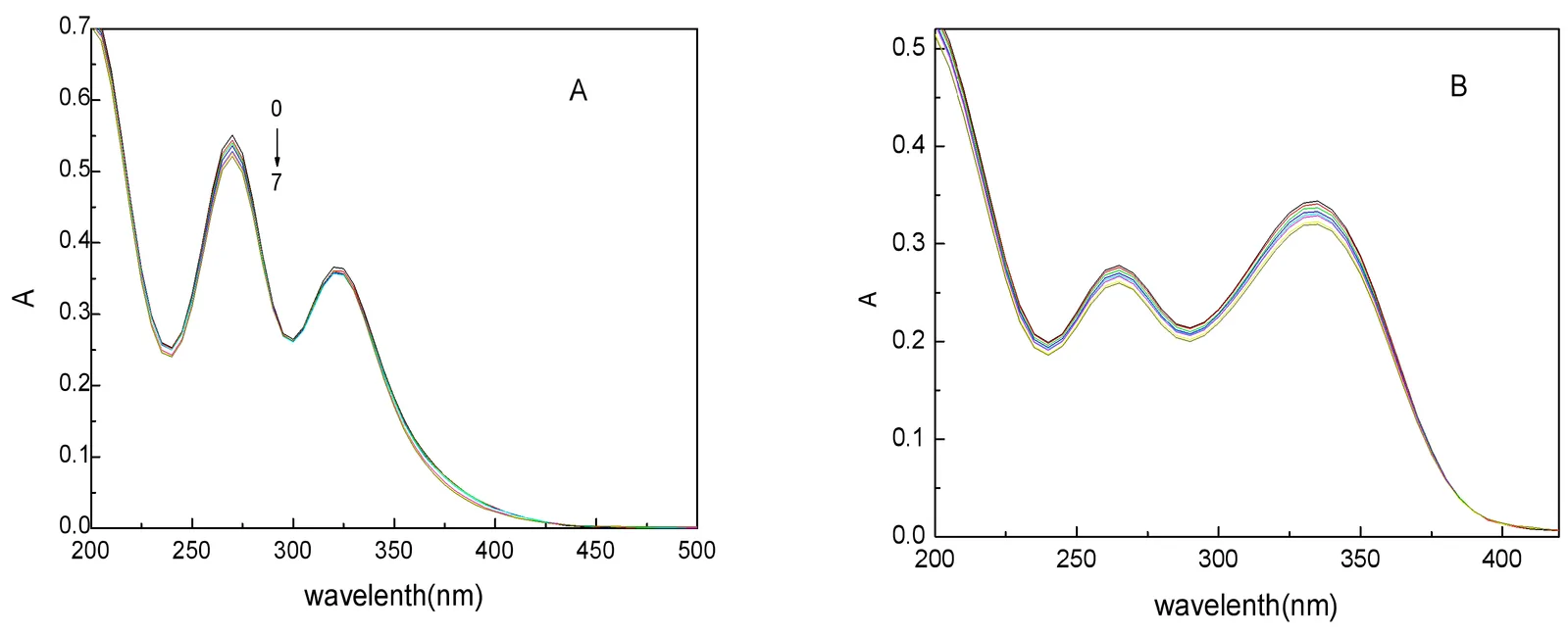
UV-Vis spectra of fixed concentration of flavonoids (2.0 × 10^−5^ M) in the presence of Co^2+^ (0.01 mol/L) of varying concentration (0 M to 8 × 10^−5^ M). (**A**) Baicalein, (**B**) Apigenin.

After interacting with Co^2+^, the spectral changes for both Bai and Api were very minimal. They mainly showed a slight hypochromic effect. There were no significant shifts or new peaks produced (Figure 4).

This indicated that the interaction between Co^2+^ and these two flavonoids was very weak. It likely formed only outer-sphere coordination complexes. Alternatively, the formation constant of the complexes was very low. This did not significantly alter the electronic transition properties of the flavonoid core. The coordination field characteristics of Co^2+^ and its low affinity for these specific flavonoid structures were the main reasons. It suggested that the formed complexes were unstable. Alternatively, their ground and excited state energy levels were similar to those of the free flavonoids.

These results systematically demonstrated that the strength and nature of flavonoid-metal interactions depended on both the type of metal ion (valence state, coordination field, softness/hardness) and the structure of the flavonoid ligand [37].

**Figure 5:**
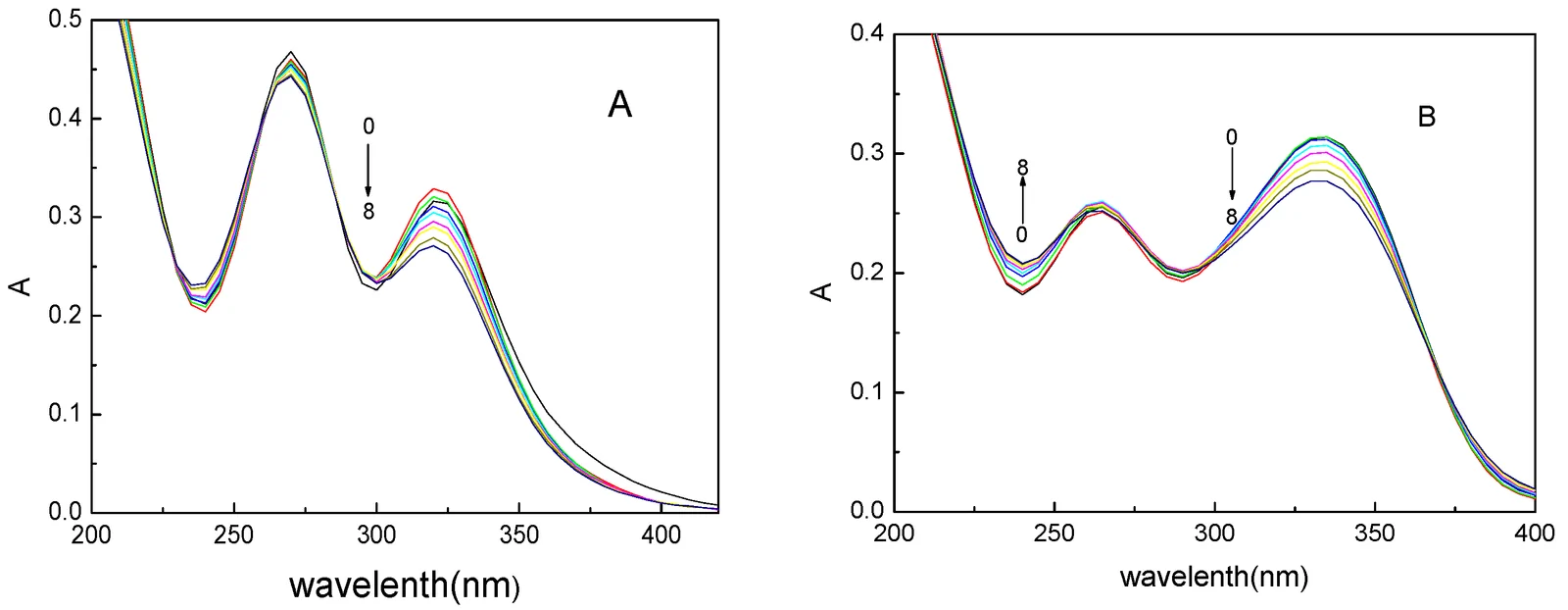
UV-Vis spectra of fixed concentration of flavonoids (2.0 × 10^−5^ M) in the presence of Ce^4+^ (0.002 mol/L) of varying concentration (2 × 10^−6^ M to 16 × 10^−6^ M). (**A**) Baicalein, (**B**) Apigenin.

Ce(IV) is a strong oxidizing agent, often used to evaluate the reducing capacity of compounds. Assessment of the antioxidant activity of Bai and Api was performed by monitoring the change in the characteristic absorption peak of Ce(IV) at 310 nm (Figure 5).

After adding Bai or Api to the Ce^4+^ solution, the intensity of the characteristic Ce^4+^ absorption peak at 317 nm decreased. The decrease in the Ce^4+^ peak intensity was both significant and concentration-dependent. This indicated that Ce^4+^ was reduced to Ce^3+^, while the flavonoids themselves were oxidized.

The decrease in absorbance directly reflected the reduction process of Ce^4+^ to Ce^3+^. The flavonoid compounds acted as electron donors and were oxidized. This experiment served as a direct spectroscopic method for evaluating the reducing capacity (antioxidant activity) of flavonoids [38].

Previous studies showed that the capacity of six flavonoids to reduce Ce(IV), represented by the slope, was in the following order: Quercetin > Kaempferol > Luteolin > Apigenin (Api) > Baicalein (Bai) > Naringenin.

This order revealed a crucial structure–activity relationship. Quercetin, with its B-ring 3′,4′-catechol group and C3-OH, exhibited the strongest activity, which aligned with the classic understanding. However, a critical finding was that the reducing capacity of Apigenin (Api) was stronger than that of Baicalein (Bai). Despite Bai possessing an A-ring catechol group, which is theoretically more easily oxidized, its experimentally measured reducing capacity was lower. This strongly suggested that in the specific experimental system (acidic sulfate-containing medium), the oxidation reaction by Ce(IV) might involve significant steric hindrance or kinetic preferences. The A-ring 5,6,7-trihydroxy structure of Bai might have a slower electron transfer rate compared to the B-ring 4′-OH of Api, possibly due to steric hindrance or its intramolecular hydrogen-bonding network. Furthermore, the oxidation potentials of different hydroxyl groups might vary in specific microenvironments. The oxidation potential of Bai’s A-ring catechol group might actually be higher than that of Api’s B-ring single phenolic hydroxyl.

This anomalous structure–activity relationship was an important discovery of this study. It indicated that the assessment of antioxidant activity must consider the specific conditions of the reaction system. This exceptional finding is a distinctive feature of your research and should be emphasized in the discussion, highlighting its deviation from conventional knowledge and the potential underlying reasons.

### 3.3. Interaction with Simplified Amphiphilic Models and pKa Shifts in a Liposomal Environment

**Figure 6:**
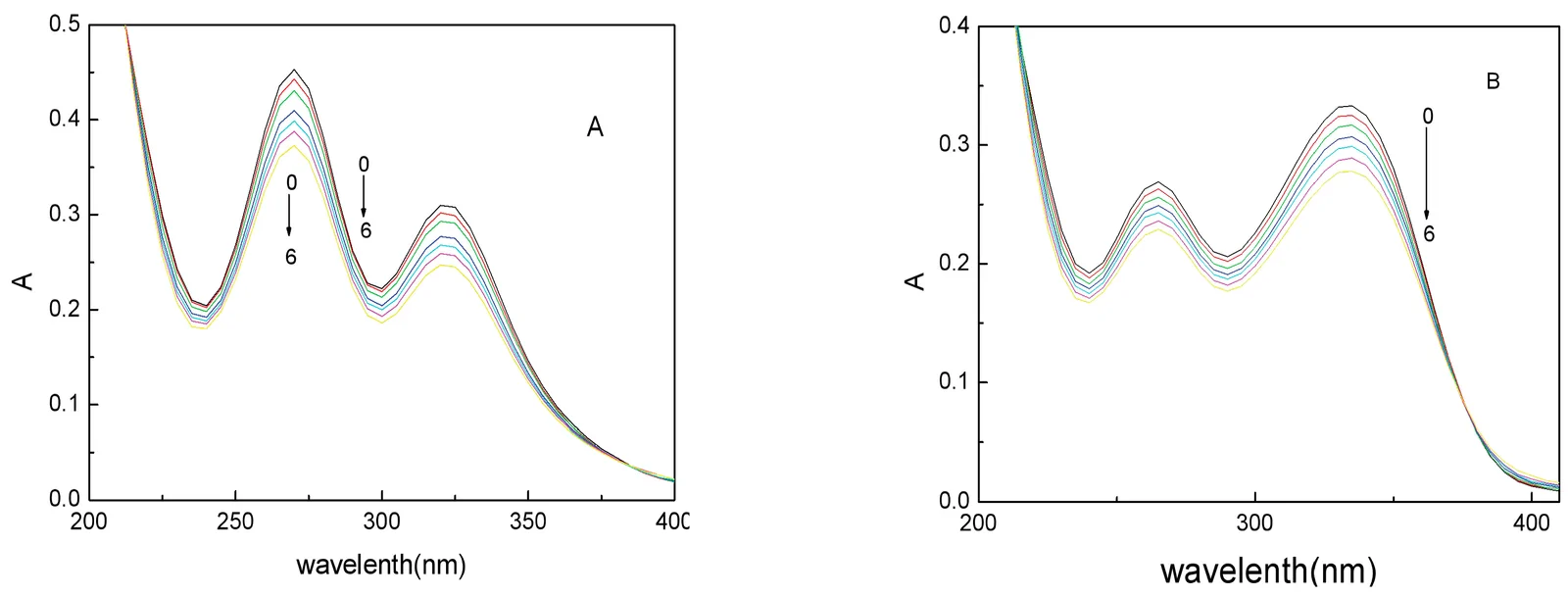
UV-Vis spectra of fixed concentration of flavonoids (2.0 × 10^−5^ M) in the presence of CTAB (0.005 mol/L) of varying concentration (5 × 10^−6^ M to 3 × 10^−5^ M). (**A**) Baicalein, (**B**) Apigenin.

**Figure 7:**
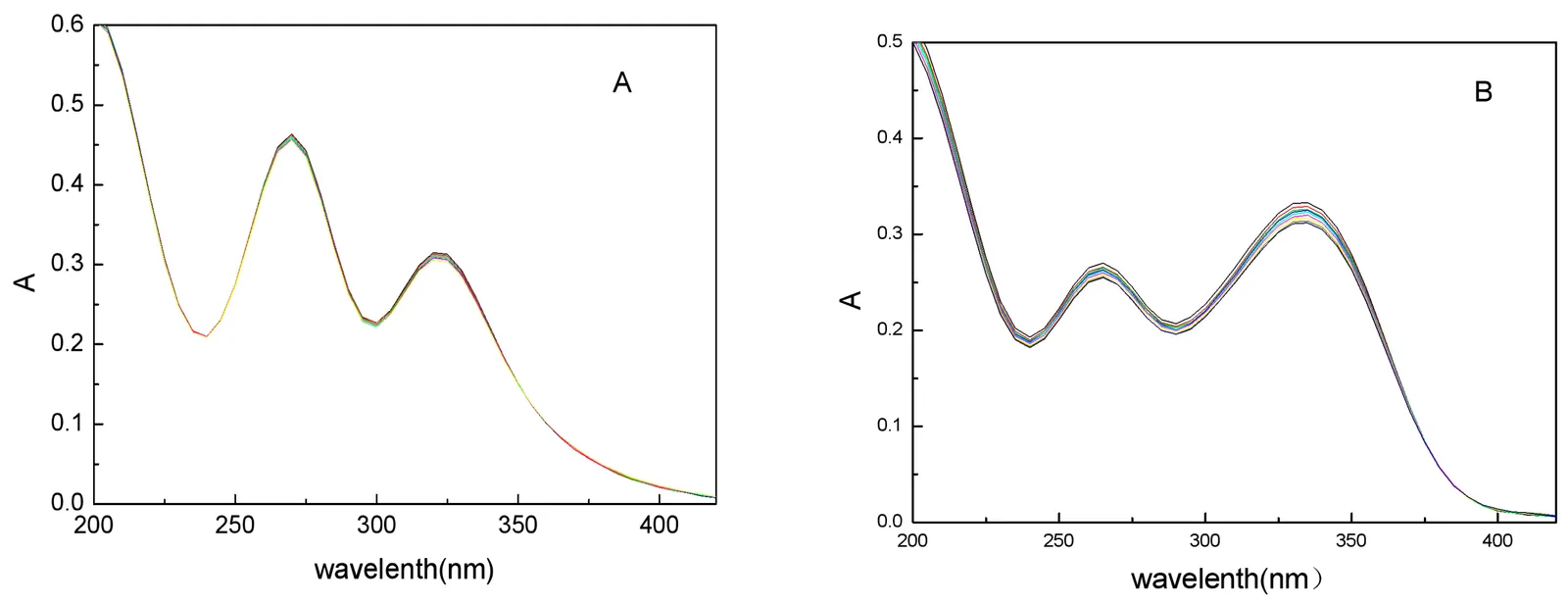
UV-Vis spectra of fixed concentration of flavonoids (2.0 × 10^−5^ M) in the presence of SDS (0.005 mol/L) of varying concentration (5 × 10^−6^ M to 3 × 10^−5^ M). (**A**) Baicalein, (**B**) Apigenin.

To simulate the interaction of flavonoids with biological membranes, their behavior with cationic (CTAB) and anionic (SDS) surfactant micelles was investigated (Figure 6 and Figure 7).

#### 3.3.1. Interaction of Baicalein and Apigenin with Cationic CTAB Micelles

After interacting with CTAB, both Bai and Api exhibited a slight red shift and hyperchromic effect in their spectra.

CTAB formed positively charged micelles. Near neutral pH, the flavonoid molecules were partially deprotonated and carried a weak negative charge. They were enriched into the Stern layer (palissade layer) of the CTAB micelles via electrostatic attraction. This micelle-water interface region possessed a lower polarity compared to bulk water, along with certain spatial confinement effects. This change in environmental conditions led to the observed red shift and hyperchromic effect in the flavonoid spectra.

#### 3.3.2. Interaction of Baicalein and Apigenin with Anionic SDS Micelles

The interaction with SDS micelles was much weaker (Figure 7). Only minor, irregular fluctuations were observed in the spectra of Bai and Api.

Mechanism Analysis: The SDS micelle surface was negatively charged. Electrostatic repulsion occurred between this surface and the similarly negatively charged flavonoid molecules. This prevented the flavonoids from entering or approaching the Stern layer of the micelles. Any interaction between the flavonoids and the SDS micelles could only occur through very weak hydrophobic forces. Consequently, almost no significant spectral changes were induced [39].

### 3.4. pH-Dependent Behavior of Flavonoid Compounds in a Biomimetic Carrier System

As shown by the measured data in the table below (Table 1), the pKa values of the flavonoid compounds differed significantly between those measured in double-distilled water alone and those measured in the presence of vesicles. After the addition of vesicles, the pKa values of all flavonoids increased (Table 1). This indicated that the proton-donating ability of the compounds decreased following their interaction with the vesicle system [40]. The influence of the membrane environment on the physicochemical properties of flavonoids was further investigated by determining their apparent pKa in liposomal/vesicle (niosome) systems. A key finding was that the pKa values of all flavonoids in the liposome/vesicle environment were higher than their corresponding values in aqueous solution, i.e., ΔpKa = pKa(membrane) − pKa(water) > 0.

The hydrophobic nature and low dielectric constant environment of the lipid bilayer greatly disfavored the stabilization of the deprotonated, negatively charged phenolate anions. From a thermodynamic perspective, the membrane environment suppressed the ionization of the phenolic hydroxyl groups. This resulted in an elevation of the apparent pKa.

**Figure 8:**
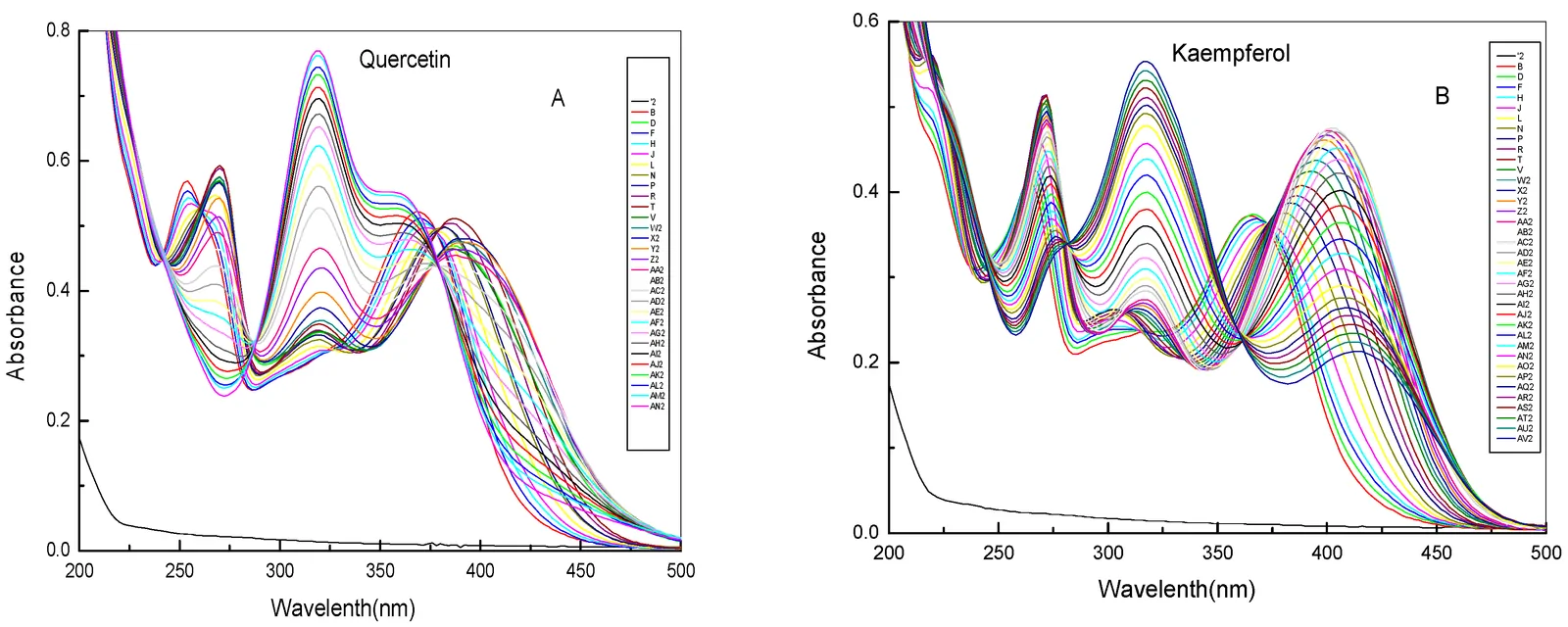
UV-Vis absorption spectra of flavonoids (2.0 × 10^−5^ M) in 5% (V%) liposome with varying NaOH concentrations (1.0 × 10^−5^ M to 3.5 × 10^−4^ M): (**A**) quercetin, (**B**) kaempferol.

In the presence of liposomes, the spectral evolution of flavonoids during deprotonation with increasing pH was observed (Figure 8). Compared to those in the aqueous phase, the titration curves of all compounds in liposomes were more gradual. Furthermore, a higher pH was required to complete the deprotonation process. This directly confirmed the aforementioned conclusion regarding the pKa shift—the membrane environment made the deprotonation of flavonoids more difficult and caused it to occur at a higher pH.

**Figure 9:**
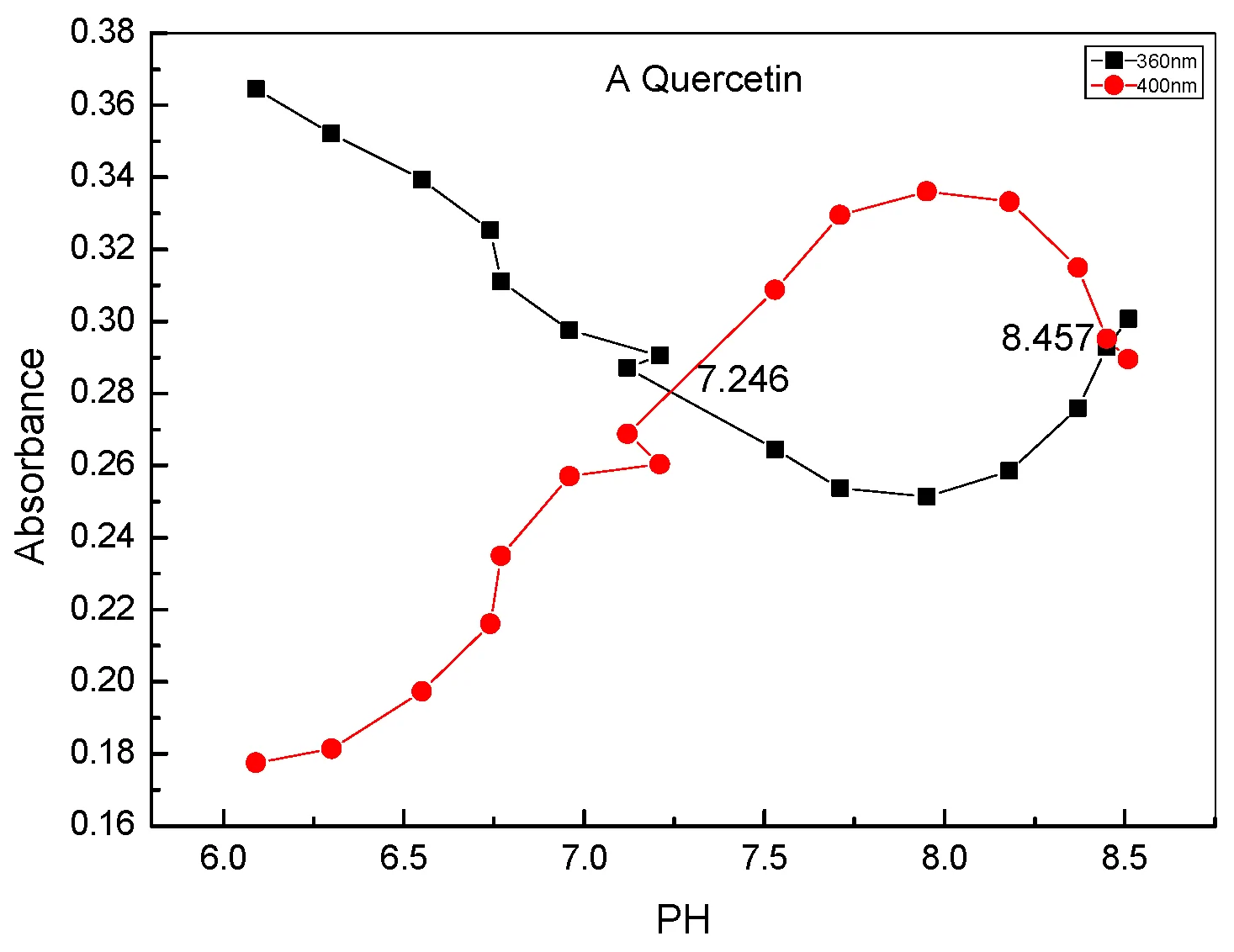
Absorbance of Quercetin at 360 nm and 400 nm in water. C_(flavonoids)_ = 2 × 10^−5^ mol/L, C_(NaOH)_ = 0.1 × 10^−5^, 2 × 10^−5^, 3 × 10^−5^, 4 × 10^−5^, 5 × 10^−5^, 6 × 10^−5^, 7 × 10^−5^, 9 × 10^−5^, 11 × 10^−5^, 13 × 10^−5^ mol/L. 15 × 10^−5^ mol/L, 17 × 10^−5^ mol/L. 19 × 10^−5^ mol/L, 21 × 10^−5^ mol/L.

**Figure 10:**
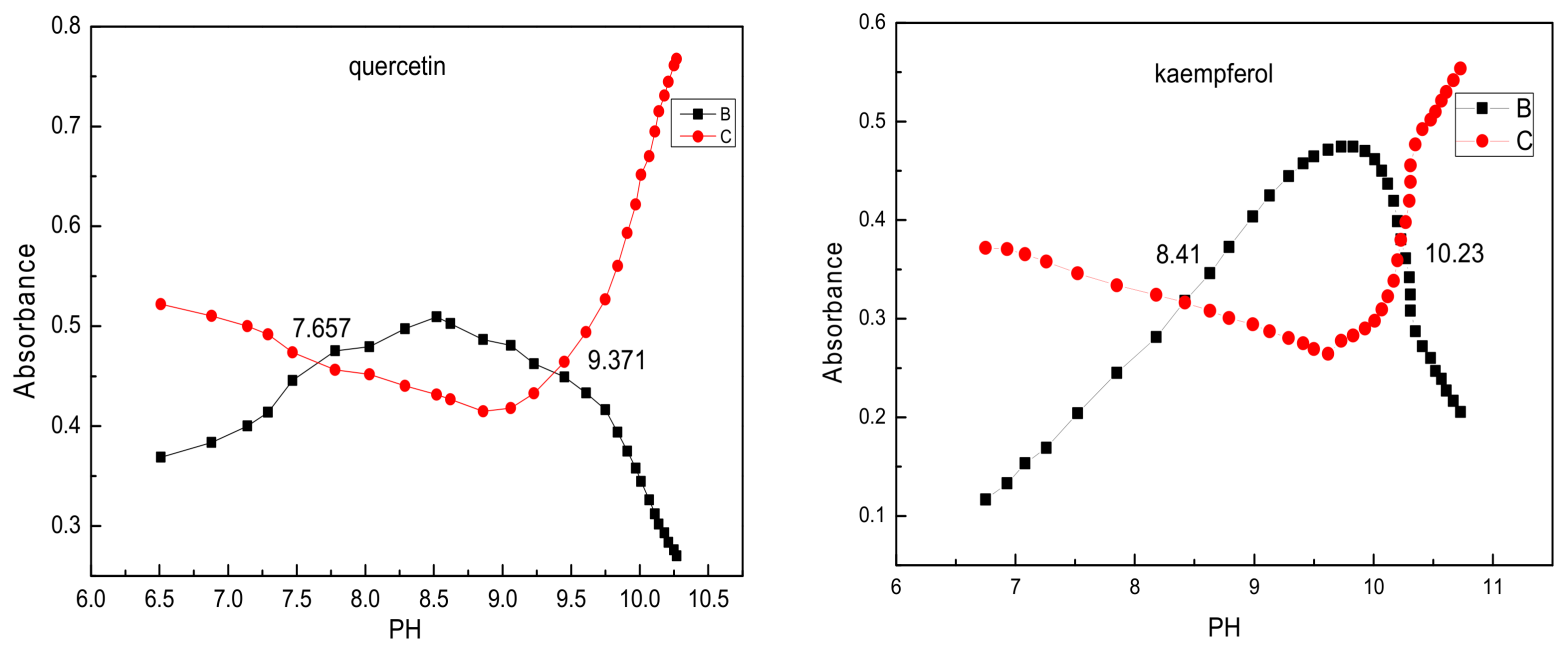
Quercetin, C_(flavonoids)_ = 2 × 10^−5^ mol/L, C_(NaOH)_ = 0.1 mol/L, range of ph: 4.22~10.28, 20 mL H_2_O + 200 µL (2 × 10^−3^ mol/L) Quercetin + 20 µL liposome + NaOH. Kempferol, C_(flavonoids)_ = 2 × 10^−5^ mol/L, C_(NaOH)_ = 0.1 mol/L, range of ph: 4.20~10.85. 20 mL H_2_O + 200 µL (2 × 10^−3^ mol/L) Kaempferol +20 µL liposome+ NaOH.

**Figure 11:**
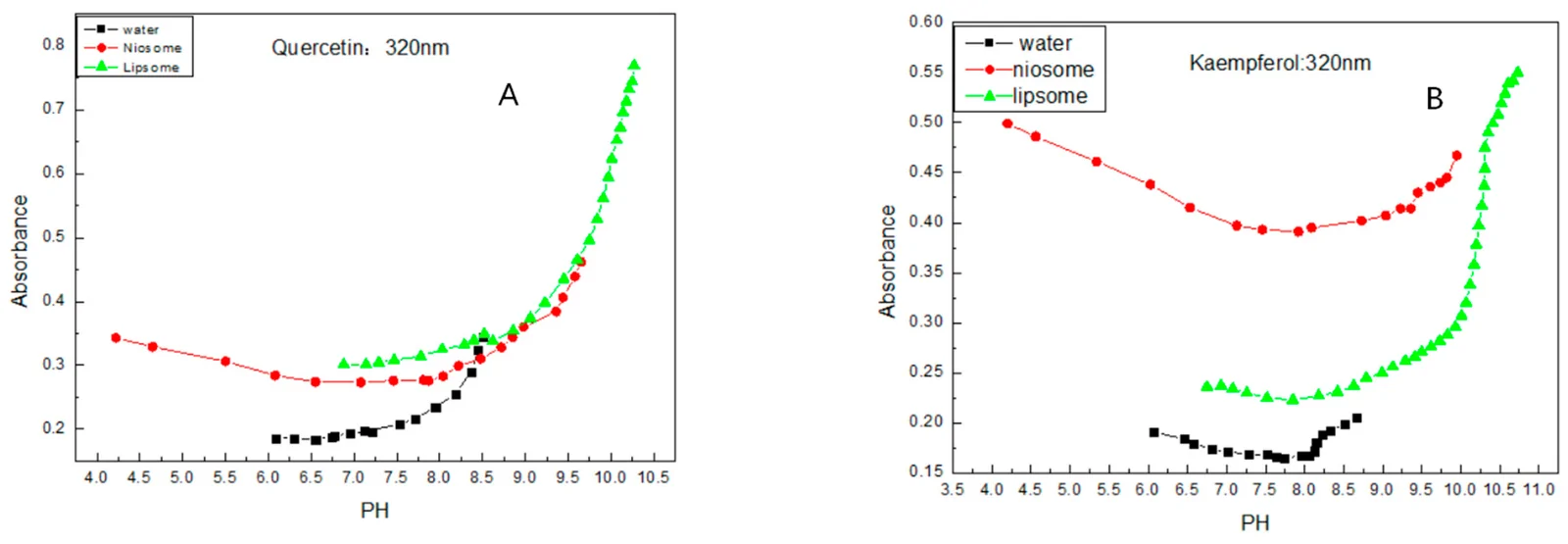
Absorbance of (**A**) Quercetin, (**B**) Kaempferol at 320 nm with fixed concentration of flavonoids (2.0 × 10^−5^ M) in a range of PH: 4.20~10.85.

From measuring the absorbance at 320 nm of Quercetin (Que) (Figure 9) and Kaempferol (Kae) in three different media (aqueous solution., niosomes, and liposomes (Figure 10)) across varying pH levels (Figure 11), the following insights were yielded. This wavelength corresponds to the characteristic absorption peak of flavonoids. Changes in absorbance directly reflect the compound’s concentration, dissolution state, and stability within the system.

(1) Protective Effect of the Carrier Systems (Solubilization and Stabilization)

The study revealed a common and significant trend: across the entire tested pH range (4.0–10.5), the absorbance of both flavonoid compounds in the niosome and liposome systems was significantly higher than that in the aqueous solution alone. This phenomenon clearly demonstrates that both niosomes (non-ionic surfactant vesicles) and liposomes (phospholipid bilayer vesicles) can effectively solubilize and encapsulate the highly hydrophobic flavonoid compounds. Both systems effectively encapsulated the hydrophobic flavonoid molecules within their hydrophobic bilayer membranes. This encapsulation transferred the flavonoid molecules from the polar aqueous phase to the non-polar membrane phase. It thereby avoided their aggregation, precipitation, or degradation in the aqueous phase. This was spectroscopically manifested as higher and more stable absorbance values [41,42].

(2) Interpretation of the “Coinciding” Behavior of Quercetin (Que): Polarity-Driven Interfacial Interaction

Quercetin exhibited consistent behavior in both carrier systems. This indicated that its interaction mode with the carrier membranes was insensitive to subtle compositional differences. Quercetin possesses a catechol structure (3′,4′-dihydroxy on the B-ring), conferring strong polarity. It serves as an excellent hydrogen bond donor and metal chelator. Based on its higher polarity, quercetin molecules were inferred to be predominantly located at the hydrophilic-hydrophobic interface of the carrier membranes. Their phenolic hydroxyl groups likely formed strong hydrogen bonds or dipole–dipole interactions with polar head groups of membrane components, such as the phosphate esters of phospholipids or the polyoxyethylene chains/hydroxyl groups of non-ionic surfactants.

Although liposomes (phospholipid bilayers) and niosomes (non-ionic surfactant monolayers) differ chemically, both provided abundant polar interaction sites for quercetin at their interface regions. This interaction was likely strong enough to dominate its behavior. Consequently, the local microenvironments created by the two carriers became functionally equivalent for quercetin. This led to the highly consistent dissociation/spectral enhancement profiles (curves), as shown in Figure 11.

(3) Interpretation of the “Diverging” Behavior of Kaempferol (Kae): Hydrophobicity-Driven Probing of the Core Region

The differential response of kaempferol to different carriers acted as a “probe signal,” revealing nanoscale physicochemical differences between the carrier membranes. Kaempferol lacks one B-ring hydroxyl group, making it significantly less polar and more hydrophobic than quercetin. It was inferred that its stronger hydrophobicity favored deeper insertion into the hydrophobic core region of the membranes, away from the interface.

Fundamental differences exist in the nature of the hydrophobic core between liposomes and niosomes. Liposomes are typically composed of phospholipids (e.g., lecithin) and cholesterol, forming a classical lipid bilayer hydrophobic region that is highly ordered, dense, and exhibits relatively low fluidity. In contrast, niosomes are formed by non-ionic surfactants, whose alkyl chains create a hydrophobic core that is generally less ordered, more loosely packed, and potentially more fluid.

Kaempferol functioned as a “hydrophobic probe”. Its specific hydrophobic microenvironment directly influenced the dissociation capability of its phenolic hydroxyl groups. In the more ordered and less polar hydrophobic core of liposomes, deprotonation likely required higher energy or was more strongly inhibited. This was manifested as differences in the slope or absolute intensity of its absorbance–pH curve compared to its behavior in niosomes, resulting in the observed curve separation. This indicates that the “quality” of the carrier membrane’s hydrophobic region is a key variable influencing the microenvironment and behavior of hydrophobic drug molecules.

This structure–activity relationship suggests that flavonoids like quercetin may possess higher physicochemical stability when traversing the alkaline environment of the upper gastrointestinal tract. It provides a key theoretical basis for predicting the oral absorption potential of flavonoids and designing more efficient delivery systems: the B-ring catechol moiety is an advantageous structural feature conferring stability across a broad pH range. Furthermore, this study strongly supports the effectiveness of liposomes and niosomes as oral delivery strategies for flavonoids.

The B-ring phenolic hydroxyl group governed the electronic effects and reducing potential. The B-ring 4′-OH of Apigenin (Api) contributed significantly to its pronounced red shift under alkaline conditions. It also contributed to Api’s relatively strong activity in the Ce(IV) reduction assay. However, a B-ring catechol group, as in Quercetin, demonstrated unparalleled advantages. These advantages were evident in stabilizing radicals and chelating metals. Api lacked an A-ring catechol group like that in Baicalein (Bai). Therefore, Api’s coordination likely occurred primarily through its B-ring 4′-OH and the C-ring C4=O carbonyl. The resulting chelates exhibited lower stability. Their conjugation extension was also limited. Consequently, this led to weaker spectral changes.

### 3.5. Influence of the Liposomal Environment on the Acid-Base Properties and Membrane-Binding Behavior of Flavonoids

Combining the data from Table 1 and Figure 10, a universal trend in pKa changes was analyzed (Table 1). Core Finding: The pKa values of all flavonoids in the liposome/niosome environment were higher than those in aqueous solution, i.e., ΔpKa > 0.

The interior of the lipid bilayer is a hydrophobic, low-dielectric-constant environment. This environment is highly unfavorable for stabilizing the negatively charged phenolate anions formed after deprotonation. From a thermodynamic perspective, the membrane environment suppressed the ionization of the phenolic hydroxyl groups, making proton release more difficult, consequently leading to an increase in the apparent pKa.

The core principle revealed by this study is as follows: Figure 11, through its clever experimental design, visualized the structure–activity relationship between a drug molecule’s physicochemical properties (polarity/hydrophobicity) and the structural heterogeneity of nanocarriers (interface region vs. hydrophobic core). It demonstrated that carriers are not inert “containers.” Instead, they can actively and differentially modulate the physicochemical state of encapsulated drugs through their nanostructure.

This analysis strongly suggested the need for quantitative validation. Techniques such as fluorescence quenching (for localization), molecular dynamics simulations (for dynamic interactions), and differential scanning calorimetry (for membrane phase behavior) should be employed. These would verify the difference in localization depth of Kaempferol within the two carrier membranes, as well as the difference in the intrinsic order of the two membrane systems themselves.

## 4. Conclusions

This study systematically investigated the structure-dependent interactions of flavonoids—specifically baicalein (A-ring ortho-dihydroxyl) and apigenin (B-ring 4′-monohydroxyl)—with metal ions (Co^2+^, Ce^4+^) and membrane–mimetic systems (CTAB/SDS micelles, liposomes, niosomes) using UV-Vis absorption spectroscopy. The results demonstrate that the protonation–deprotonation behavior of phenolic hydroxyl groups, and thus the electronic properties of flavonoids, are highly sensitive to pH. Under weakly acidic conditions (H_2_SO_4_ ≤ 5.0 × 10^−5^ mol/L), no significant spectral changes were observed for either compound, indicating that the conjugated structure and electronic transitions of the flavonoid molecules were not affected within this acidity range. In contrast, under alkaline conditions, both compounds exhibited pronounced red shifts upon deprotonation, with apigenin showing a larger spectral shift, highlighting the major contribution of the B-ring hydroxyl to electronic delocalization.

Metal chelation was highly structure-dependent. Co^2+^ formed only weak outer-sphere complexes with both baicalein and apigenin, whereas the Ce^4+^ reduction assay revealed an unexpected structure–activity relationship: apigenin (B-ring 4′-monohydroxyl) exhibited a stronger reducing capacity than baicalein (A-ring ortho-dihydroxyl) in the acidic sulfate-containing medium. This finding challenges the classical view that ortho-dihydroxyl groups always confer superior antioxidant activity, and instead highlights the critical roles of reaction medium, steric accessibility, and kinetic factors in modulating electron transfer pathways.

In membrane–mimetic systems, cationic CTAB micelles induced significant spectral changes due to electrostatic enrichment, whereas anionic SDS micelles showed minimal interaction due to electrostatic repulsion. Incorporation into liposomes and niosomes uniformly elevated the apparent pKa values of the tested flavonoids (ΔpKa > 0), demonstrating that the hydrophobic, low-dielectric environment of the lipid bilayer suppresses ionization and stabilizes the neutral form. A comparative analysis of quercetin and kaempferol revealed that the highly polar quercetin localizes at the membrane interface, exhibiting consistent behavior across different carriers, whereas the more hydrophobic kaempferol partitions more deeply into the bilayer core, thereby probing distinct physicochemical properties of liposomes versus niosomes.

Collectively, these findings establish a direct relationship between specific flavonoid structural features—such as B-ring catechol versus monohydroxyl substitution and overall polarity—and their behavior in biomimetic environments. This work provides a quantitative and mechanistic basis for the structure-guided design of flavonoid-loaded nanocarriers, with direct implications for improving the bioavailability, stability, and targeted antioxidant delivery of these promising natural products.

## Figures and Tables

**Table 1:** pKa Comparison of Flavonoids (Partial data from Ref. [40]).

	Quercetin			Kaempferol		
	NaOH	niosome	Lipsome	NaOH	niosome	Lipsome
Pka1	7.264	--------	7.657	-------	--------	8.41
Pka2	8.457	9.329	9.371	8.089	8.967	10.23

## Data Availability

The original contributions presented in the study are included in the article. Further inquiries can be directed to the corresponding author.
